# Pulmonary Embolism with Abdominal Pain and ST Elevation: A Case Report

**Published:** 2014-07

**Authors:** Mohammad Javad Fallahi, Seyed Masoom Masoompour, Mehdi Mirzaee

**Affiliations:** Pulmonary and Critical Care Division, Department of Internal Medicine, Nemazee Teaching Hospital, Shiraz University of Medical Sciences, Shiraz, Iran

**Keywords:** Pulmonary embolism, Abdominal pain, Electrocardiography

## Abstract

Pulmonary embolism is considered as a great masquerader due to its frequent nonspecific signs and symptoms. Typically pulmonary embolism is under-diagnosed or over-diagnosed. In this study a patient with pulmonary embolism is reported in which the patient exhibited two unusual manifestations namely; right upper quadrant abdominal pain and ST-T elevation in anterior precordial leads. Due to the fact that the patient did not display typical pulmonary embolism symptoms and its major risk factors, extensive workup to discern the cause was carried out. The examination included abdominal sonography, kidney ureter and bladder Computed Tomography scan (CT-scan) and coronary angiography. Eventually after a six-day delay, pulmonary embolism was diagnosed by spiral chest CT scan. This case and several other similar reports underlines the fact that while various other common causes may exist for right upper abdominal pain, one should always consider pulmonary embolism as a possible cause especially when backed up with ECG finding.

## Introduction

Pulmonary embolism is considered as a great masquerader due to its frequent nonspecific signs and symptoms. One-day and seven-days mortality after pulmonary embolism alone may be as high as 27% and 40% respectively.^1^ Consequently, a timely diagnosis could be a lifesaving matter for many patients. Physicians should consider pulmonary embolism in any patient that exhibits new onset dyspnea, pleuritic chest pain, hemoptysis, and/or unexplained hypotension or syncope.^2^ However, diagnosis is typically confirmed in only 20% of patients with suspected pulmonary embolism.^3^ Occasionally, as in the case of this report, pulmonary embolism can cause unusual clinical manifestation. This study presents a case of pulmonary embolism where atypical clinical and electrocardiographic presentation led to an array of invasive and noninvasive diagnostic work up and hazardous delay in diagnosis.

## Case Report

**Figure 1:**
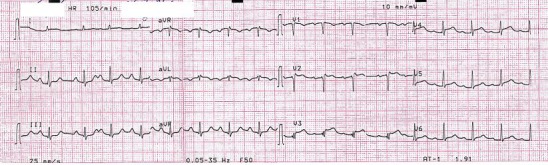
The patient’s ECG shows ST-T segment elevation in lead I, AVL and V1-V4.

**Figure 2:**
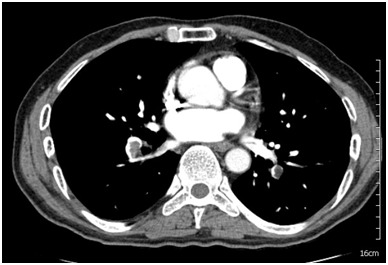
Spiral chest CT scan demonstrates bilateral filling defects of pulmonary arteries.

A 54-year-old man, heavy smoker, was awakened in an early morning due to sudden onset of right upper quadrant abdominal pain. This occurred six days prior to his admission in a ward. His pain aggravated with respiration which was initially associated with cold sweating and dyspnea. The patient did not report any cough or hemoptysis. Referring to a local emergency room, the on-call physician requested work-ups including Electrocardiogram (ECG), chest radiography and abdominal sonography. The only abnormal finding was sinus tachycardia. The patient was sent home with antacid medication after which no improvement was observed. The same afternoon, a gastroenterologist performed and upper endoscopic study turned to be normal. The results of liver function test, amylase and cardiac troponin were also normal except for a mild AST elevation and high troponin level (665 pg/ml [NL: 0-140]). The second ECG revealed ST-T segment elevation in lead I, Avl and v1-v4 (figure 1). The patient was then admitted to the coronary care unit of another hospital. The following morning, coronary angiography was carried out that only exposed mild sluggish flow in diagonal 2 (D2) branch of coronary artery. A day later the patient was released with his consent but returned a couple of days later with the recurrence of the right upper quadrant (RUQ) abdominal pain combined with the right flank pain. Following the re-admission process in the same hospital, his symptoms were misinterpreted as the renal colic. A second abdominal sonography and kidney ureter bladder CT scan without contrast was requested that turned to be normal. Ultimately the patient was referred and admitted to our hospital after which he complained of RUQ abdominal pain being aggravated by respiration. He had neither significant medical history nor major risk factors for venous thromboembolism. Vital signs on admission were pulse rate at 110 beat per minute, respiratory rate at 20 breaths per minute, blood pressure at 100/70 mmHg  and body temperature at 37.2°C. Additionally, no tenderness was observed during abdominal examination and various other aspects of physical examination including lungs were normal. Furthermore, no evidence of deep venous thrombosis in his extremities was noted. Arterial blood gas analysis revealed PH at 7.49, Pco2 at 34.1, HCO3 at 26.4, PaO2 at 56.3 mmHg and oxygen saturation while breathing the ambient air at 92%.  ECG exhibited normal sinus rhythm, residual ST elevation and T wave inversion in v1-v2 leads. Based on the arterial hypoxemia and ECG changes, suspicion was raised for pulmonary embolism issue. Spiral chest CT-scan was performed that exposed bilateral pulmonary embolism (figure 2). In order to do risk stratification for approach to pulmonary embolism, transthoracic echocardiography was carried out but displayed no evidence of the right ventricular dysfunction. The patient was then prescribed with heparin anticoagulation followed by warfarin. A work-up for exploring possible underlying malignancy including complete blood count, erythrocyte sedimentation rate, urine analysis, stool exam, prostate specific antigen and abdominal and pelvic CT scan was unremarkable. The patient was then discharged on warfarin.

## Discussion

Pulmonary embolism is potentially a fatal disease being frequently under-diagnosed or over-diagnosed. There are several prediction models that estimate pretest probability of pulmonary embolism such as wells score or Geneva score. Nonetheless, pulmonary embolism may exhibit itself in such unusual clinical manner that clinicians essentially do not consider pulmonary embolism as a possible diagnosis.

The case described here, presents two unsuspected findings for pulmonary embolism: RUQ abdominal pain and ST-T elevation in precordial leads. These findings could prevent extensive diagnostic work-ups and hazardous delays in judgment. Ultimately, pulmonary embolism in this case was suspected based on arterial hypoxemia.

There are several case reports that describe varied combination of abdominal pain and flank pain as a presenting or associated symptom of pulmonary embolism.^4^^-^^6^ In a case series,^5^ authors described three cases of pulmonary embolism that presented with flank pain. The first one was a 52-year-old man with past history of Hodgkin’s lymphoma who presented with several days of right abdominal and flank pain. The author reported that the patient also had positive Murphy’s sign. Diagnosis of pulmonary embolism was suspected based on the right lower lobe infiltrations of lung which appeared in the initial cuts of abdominal CT scan and occurrence of hemoptysis. In comparison with this case, our patient had not had major risk factors of pulmonary embolism such as immobility, surgery, known cancer or previous thromboembolic disease. As a rare and minor risk factor for deep vein thrombosis, coronary angiography should be considered, however the symptom in current case was presented before coronary angiography and the patient did not have any size difference in his lower extremities. Considering the above mentioned explanations the first treating physician did not consider color doppler sonography of lower extremities for the patient.

In a recent report^7^ on a young man, three days after left-leg trauma, presented with abdominal pain, dyspnea and ST-T elevation in v1-v4 electrocardiogram. Following cardiopulmonary arrest, bilateral massive pulmonary embolism was documented in pulmonary angiography. Subsequent autopsy confirmed large clot in main pulmonary arteries and normal coronary arteries.

The mechanism of abdominal pain in pulmonary embolism is not well known as it could be due to diaphragmatic pleurisy or liver congestion. 

Pulmonary embolism has protean ECG manifestations.  Sinus tachycardia and non-specific ST-T change are among the most common findings. New onset atrial fibrillation, right bundle branch block blocks and S_1_Q_3_T_3 _pattern also was seen.^8^ ST-T elevation in anterior precordial is a rare ECG finding of pulmonary embolism. The exact mechanism of this ECG change is not clear, though the most stated possibility is paradoxical coronary embolism via patent foramen ovale.^9^

In a patient with chest pain or other symptom suggestive of myocardial ischemia, ST-T elevation in conjunction with abnormal troponin level may lead clinicians to consider myocardial infarction as the utmost probability. It can lead to performing coronary angiography as in our case.^10^ After ruling out the possibility of coronary artery disease, the elevated level of troponin could be considered as a sign of pulmonary embolism. In the first admission, one day after angiography, the patient left the hospital before the treating physician recommends discharge, thus echocardiography was not performed at that time. During the second admission, echocardiography was done to stratify the patient’s level of risk and subsequently selecting appropriate therapeutic approach.

The interesting features of our patient were simultaneous occurrence of two unusual manifestation of pulmonary embolism in a person without major risk factors. In addition, the patient did not report any other symptom of pulmonary embolism such as cough or hemoptysis. 

Although there are several more common causes of right upper abdominal pain, pulmonary embolism should be considered as a possible diagnosis especially when supporting ECG findings and/or abnormal troponin exists.
